# Correction: Synthesis, docking and biological evaluation of purine-5-*N*-isosteres as anti-inflammatory agents

**DOI:** 10.1039/d4ra90072c

**Published:** 2024-07-12

**Authors:** Ahmed M. El-Saghier, Souhaila S. Enaili, Aly Abdou, Amany M. Hamed, Asmaa M. Kadry

**Affiliations:** a Chemistry Department, Faculty of Science, Sohag University Sohag 282524 Egypt el.saghier@science.sohag.edu.eg; b Chemistry Department, Faculty of Science, Al Zawiya University Al Zawiya Libya

## Abstract

Correction for ‘Synthesis, docking and biological evaluation of purine-5-*N*-isosteres as anti-inflammatory agents’ by Ahmed M. El-Saghier *et al.*, *RSC Adv.*, 2024, **14**, 17785–17800, https://doi.org/10.1039/D4RA02970D.

The authors regret that the title shown in the original article was incorrect. The correct title is as shown above. The authors also regret the omission of a funding acknowledgement in the original article. This acknowledgement is given below.

The authors also thank Magda H. Abdellattif and Taif University Researchers Supporting Project Number TURSP-2020/91, Taif University, Saudi Arabia.

The Royal Society of Chemistry apologises for these errors and any consequent inconvenience to authors and readers.

